# Fusion mechanism of 2019-nCoV and fusion inhibitors targeting HR1 domain in spike protein

**DOI:** 10.1038/s41423-020-0374-2

**Published:** 2020-02-11

**Authors:** Shuai Xia, Yun Zhu, Meiqin Liu, Qiaoshuai Lan, Wei Xu, Yanling Wu, Tianlei Ying, Shuwen Liu, Zhengli Shi, Shibo Jiang, Lu Lu

**Affiliations:** 1 Key Laboratory of Medical Molecular Virology (MOE/NHC/CAMS), School of Basic Medical Sciences, Fudan-Jinbo Joint Research Center, Fudan University, Shanghai, China; 2 National Laboratory of Biomacromolecules, Institute of Biophysics, Chinese Academy of Sciences, Beijing, China; 3 CAS Key Laboratory of Special Pathogens and Biosafety, Wuhan Institute of Virology, Chinese Academy of Sciences, Wuhan, China; 4 Guangdong Provincial Key Laboratory of New Drug Screening, Guangzhou Key Laboratory of Drug Research for Emerging Virus Prevention and Treatment, School of Pharmaceutical Sciences, Southern Medical University, Guangzhou, China; 5 Lindsley F. Kimball Research Institute, New York Blood Center, New York, NY USA

**Keywords:** Target identification, Mechanisms of disease

Very recently, a novel coronavirus, 2019-nCoV, emerged in Wuhan, China and then quickly spread worldwide, resulting in >17,388 confirmed cases and 361 deaths as of 3 February 2020, thus calling for the development of safe and effective therapeutics and prophylatics.^1,2^

Similar to severe acute respiratory syndrome (SARS)-CoV, 2019-nCoV belongs to lineage B betacoronavirus, and it has the ability to utilize human angiotensin-converting enzyme 2 (ACE2) as a receptor to infect human cells.^3^ SARS-CoV spike (S) protein S2 subunit plays a key role in mediating virus fusion with and entry into the host cell, in which the heptad repeat 1 (HR1) and heptad repeat 2 (HR2) can interact to form six-helical bundle (6-HB), thereby bringing viral and cellular membranes in close proximity for fusion. Using S-HR1 as a target, we have previously designed and developed several potent fusion inhibitors against SARS-CoV (e.g., SARS-HR2P)^4^ and Middle East respiratory syndrome (MERS)-CoV (e.g., MERS-HR2P).^5^ However, it is unclear whether 2019-nCoV also possesses a similar fusion and entry mechanism as that of SARS-CoV and MERS-CoV, and if so, whether a 2019-nCoV S-HR1 can also serve as an important target for the development of 2019-nCoV fusion/entry inhibitors.

**Fig. 1:**
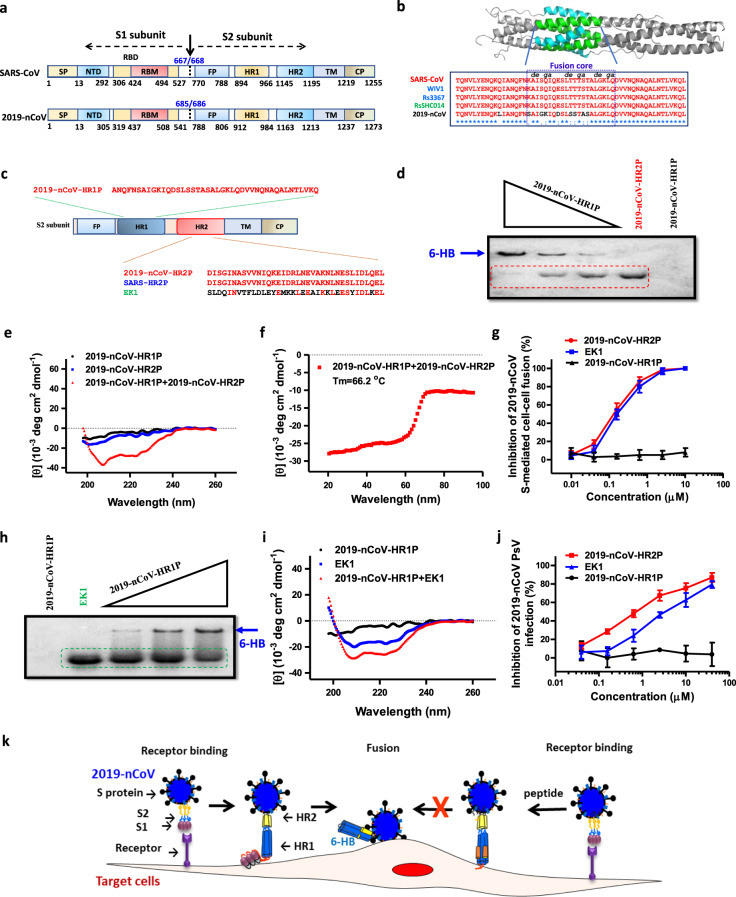
Study of the fusion mechanism of 2019-nCoV and characterization of a fusion inhibitor derived from the HR2 domain in spike protein of 2019-nCoV and a pan-CoV fusion inhibitor. **a** Schematic representation of the 2019-nCoV S protein. SP signal peptide, RBD receptor-binding domain, RBM receptor-binding motif, FP fusion peptide, HR1 heptad repeat 1, HR2 heptad repeat 2, TM transmembrane domain, CP cytoplasm domain. The residue numbers of each region correspond to their positions in S protein of SARS-CoV and 2019-nCoV, respectively. **b** The sequence alignment of HR1 core domains in SARS-CoV, SL-CoVs, and 2019-nCoV. **c** Sequences of 2019-nCoV-HR1P, 2019-nCoV-HR2P, SARS-HR2P, and EK1. **d** Determination of the interactions between 2019-nCoV-HR1P and 2019-nCoV-HR2P. Bands of 2019-nCoV-HR2P are highlighted in red box; the blue arrows indicate the bands of 6-HB. **e** Circular dichroism (CD) spectra of 2019-nCoV-HR1P, 2019-nCoV-HR2P, and 2019-nCoV-HR1P/2019-nCoV-HR2P complex. **f** Melting curves of the 2019-nCoV-HR1P/2019-nCoV-HR2P complex. **g** Inhibitory activity of peptides on 2019-nCoV S-mediated cell–cell fusion. **h** Determination of the interactions between 2019-nCoV-HR1P and EK1. Bands of EK1 are highlighted in green box; the blue arrows indicate the bands of 6-HB. **i** CD spectra of 2019-nCoV-HR1P, EK1, and 2019-nCoV-HR1P/EK1 complex. **j** Inhibition of peptides on pseudotyped 2019-nCoV infection. **k** The putative antiviral mechanism of 2019-nCoV-HR2P and EK1. After binding of RBD in S1 subunit of 2019-nCoV S protein to the potential receptor ACE2 on the host cell, S2 subunit changes conformation by inserting FP into the cell membranes and triggering the association between the HR1 and HR2 domains to form 6-HB, which brings the viral and cellular membranes in close proximity for fusion (left part of **k**). In the presence of 2019-nCoV-HR2P or EK1 peptide, three copies of the peptide bind to the 2019-nCoV S-HR1-trimer to form heterologous 6-HB, thus blocking the formation of viral homologous 6-HB and thus inhibiting viral and cell membrane fusion (right part of **k**)

Through amino acid (aa) sequence alignment with SARS-CoV and 2019-nCoV S protein,^6,7^ we located the functional domain in 2019-nCoV S protein, including N-terminal domain (aa14–305), receptor-binding domain (aa319–541), and receptor-binding motif (aa437–508) in S1 subunit (aa14–685) and fusion peptide (aa788–806), HR1 (aa912–984), HR2 (aa1163–1213), transmembrane domain (aa1214–1237) and cytoplasm domain (aa1238–1273) in S2 subunit (aa686–1273) (Fig. 1a).

In the post-fusion hairpin conformation of the SARS-CoV or MERS-CoV S protein, the HR2 domain forms both rigid helix and flexible loop to interact with HR1 domain (Fig. 1b). There are many strong interactions between HR1 and HR2 domains inside the helical region, which is thus designated “fusion core region” (HR1_core_ and HR2_core_ regions, respectively). According to the sequence alignment, the 2019-nCoV and SARS-CoV S2 subunits are highly conserved, with 92.6% and 100% overall identity in HR1 and HR2 domains, respectively. However, inside the HR1_core_ region, 8 of the 21 residues show mutation (~38% difference). This is significantly different from the HR1_core_ region of previously identified SARS-like viruses, such as WIV1, Rs3367, and RsSHC014, which are 100% identical to that of SARS-CoV (Fig. 1b). These novel point mutations in 2019-nCoV S2 subunit may change the interaction pattern between HR1 and HR2 domains in the post-fusion core, thus affecting the 6-HB formation. Based on our previous experience, we have now designed HR1- and HR2-derived peptides, designated 2019-nCoV-HR1P (aa924–965) and 2019-nCoV-HR2P (aa1168–1203), respectively (Fig. 1c), and explored their biological characteristics. Since the 2019-nCoV and SARS-CoV S-HR2 sequences are 100% identical, 2019-nCoV-HR2P may act as a fusion inhibitor in much the same way as our reported SARS-CoV fusion inhibitor, SARS-HR2P.^4^ Under native electrophoresis as described before,^4^ 2019-nCoV-HR2P, which carries negative charges, moved down to a lower gel position, while 2019-nCoV-HR1P, which carries positive charges, moved up and off the gel (Fig. 1d) in a manner similar to SARS-CoV-HR1P^4^ in native-polyacrylamide gel electrophoresis (PAGE) assay. Notably, in the 2019-nCoV-HR1P/2019-nCoV-HR2P mixture, new bands emerged at the upper part in the native-PAGE gel in a 2019-nCoV-HR1P dose-dependent manner, indicating that 2019-nCoV-HR2P could interact with 2019-nCoV-HR1P to form a complex, possibly 6-HB. We then assessed the secondary structures of 2019-nCoV-HR1P, 2019-nCoV-HR2P, and 2019-nCoV-HR1P/2019-nCoV-HR2P complex, using circular dichroism as previously described.^4^ While 2019-nCoV-HR1P alone and 2019-nCoV-HR2P alone exhibited low helicity (<30%), the 2019-nCoV-HR1P/2019-nCoV-HR2P complex exhibited the characteristic helicity of 6-HB, with minimum values at 208 and 222 nm and helicity of 84.4% (Fig. 1e). Moreover, the 2019-nCoV-HR1P/2019-nCoV-HR2P complex showed good thermal stability with *T*_m_ of 66.2 ^o^C (Fig. 1f).

These results confirm, for the first time, that 2019-nCoV HR1 and HR2 regions are able to interact with each other to form 6-HB and suggest that 2019-nCoV-HR2P may inhibit 2019-nCoV fusion with and entry into the target cell, as we showed before with SARS-CoV, MERS-CoV, and other human CoVs.^4,5,8^ To confirm this hypothesis, we herein developed a 2019-nCoV S-mediated cell–cell fusion assay as previously described.^4,5,8^ Using this assay, we demonstrated that 2019-nCoV-HR2P exhibited potent fusion-inhibitory activity with a half maximal inhibitory concentration (IC_50_) of 0.18 µM (Fig. 1g), indicating that the 2019-nCoV HR1 region could serve as an ideal target site. On the other hand, 2019-nCoV-HR1P exhibited no significant inhibitory effect at concentrations up to 40 µM, consistent with other coronavirus HR1-derived peptides, such as SARS-HR1P and MERS-HR1P.^5,8^

We previously reported that the HR1 region in various coronaviruses is a conserved target site, and based on that evidence, we designed a pan-coronavirus fusion inhibitor, denoted as EK1.^8^ Compared with 2019-nCoV-HR2P, EK1 shows significant sequence variation, but interestingly, EK1 could also bind 2019-nCoV-HR1P in native-PAGE in a manner similar to that of 2019-nCoV-HR2P (Fig. 1h). The 2019-nCoV-HR1P/EK1 complex also showed high helicity (77.9%) (Fig. 1i). EK1 also exhibited effective fusion inhibitory activity with IC_50_ of 0.19 µM (Fig. 1g). Taken together, these results suggest that the 2019-nCoV S-HR1 region is also a promising conserved target for developing effective CoV fusion/entry inhibitors.

Taking still another step forward, we assessed the inhibitory effect of 2019-nCoV-HR2P or EK1 on 2019-nCoV pseudovirus infection in ACE2-expressing 293T cells. As shown in Fig. 1j, both 2019-nCoV-HR2P and EK1 peptides could significantly inhibit 2019-nCoV pseudovirus infection in a dose-dependent manner with an IC_50_ values of 0.98 and 2.38 µM, respectively.

Collectively, then, we have confirmed that 2019-nCoV S-HR1 and S-HR2 play key roles in mediating 2019-nCoV fusion with and entry into the host cell. 2019-nCoV may have similar membrane fusion mechanism as that of SARS-CoV. When S1 protein recognizes its receptor on human cells, the HR1 and HR2 domains are exposed to interact with each other, forming 6-HB to mediate membrane fusion between virus and target cell (Fig. 1k). Notably, both 2019-nCoV-HR2P and EK1, the pan-CoV fusion inhibitor, exhibited potent inhibitory activity against S-mediated cell–cell fusion and 2019-nCoV pseudovirus infection, suggesting potential development of either 2019-nCoV-HR2P or EK1 peptide in nasal spray and inhalation formulations, respectively, to prevent and treat 2019-nCoV infection.
